# Peritumoral Edema and Subcortical Tumor Location in Glioblastoma Outcome Prediction: An Automated Analysis of Radiological and Topographical Features

**DOI:** 10.3390/cancers18152413

**Published:** 2026-07-27

**Authors:** Anton Stenwall, Jesper Nillius, Joao M. Sousa, David Bouget, Markus Fahlström, Johan Wikström, Francesco Latini

**Affiliations:** 1 Neurosurgery, Department of Medical Sciences, Uppsala University, 75185 Uppsala, Swedenfrancesco.latini@neuro.uu.se (F.L.); 2 Department of Surgical Sciences, Molecular Imaging and Medical Physics, Uppsala University, 75185 Uppsala, Sweden; 3 Department of Health Research, SINTEF Digital, 7034 Trondheim, Norway; david.bouget@sintef.no; 4 Neuroradiology, Department of Surgical Sciences, Uppsala University, 75185 Uppsala, Sweden

**Keywords:** glioblastoma, Brain-Grid, peritumoral edema, survival, neuroimaging, automated segmentation

## Abstract

**Background**: Glioblastoma (GBM) shows marked heterogeneity in overall survival (OS), yet robust radiological prognostic markers remain limited. The prognostic value of peritumoral edema and tumor location remains uncertain. **Objective**: To evaluate whether peritumoral edema, tumor burden, and spatial tumor distribution predict OS using automated MRI analysis and Brain-Grid-based topographical mapping. **Methods**: In this retrospective study, preoperative T1-contrast-enhanced and T2-FLAIR MRI sequences from 271 patients with IDH-wildtype GBM were analyzed using automated segmentation (Raidionics). Tumor and edema volumes, edema-to-tumor ratio (ETR), and voxel-wise infiltration patterns were extracted. Location was mapped using the Brain-Grid system. Survival was assessed using Kaplan–Meier and multivariable Cox regression adjusted for age and tumor volume, with correction for multiple testing. **Results**: In multivariable analysis, only age remained a conventional independent predictor of OS (HR 1.03, *p* < 0.001). Tumor volume, edema volume, and ETR were not associated with survival. In contrast, Brain-Grid analysis identified two centrally located subcortical voxel regions that remained significantly associated with shorter OS after full adjustment and multiple testing correction. Total number of infiltrated voxels showed no prognostic value. **Conclusions**: Spatially defined subcortical infiltration patterns, rather than global tumor or edema burden, independently stratify survival in GBM. These findings highlight the prognostic relevance of voxel-level tumor topography and support further validation of Brain-Grid-based imaging biomarkers.

## Simple Summary

Glioblastoma is the most common and aggressive primary brain cancer in adults, but patients with similar tumors often have markedly different outcomes. While tumor size is routinely assessed, conventional anatomical classifications do not account for involvement of important subcortical brain structures that limit safe surgical removal and support important brain functions. In this study, we used an automated imaging analysis method to measure tumor size, changes in the surrounding brain tissue, and precise tumor location in a large cohort of patients with glioblastoma. We found that tumor infiltration of two specific deep brain regions was associated with shorter survival, whereas tumor size relative to changes in the surrounding brain tissue was not. These findings suggest that detailed mapping of tumor location may provide additional prognostic information beyond conventional imaging and could contribute to improved preoperative assessment and future personalized treatment strategies.

## 1. Introduction

Glioblastoma is a malignant brain tumor known for being diffusely infiltrative, having a therapy resistant nature and an overall poor prognosis. Despite multimodal standard treatment regimes, the median survival is around 15 months. Though the prognosis is generally poor there is a large variety in survival ranging from weeks to multiple years [1,2]. This heterogeneous course exerts substantial stress for families and poses a clinical challenge for healthcare providers. Methods to predict outcome have been highly requested in the neurooncological and neurosurgical field with contrasting results, especially regarding radiological features [3,4,5].

Much focus in previous studies has been directed towards the volume of the tumor and the surrounding edema. Several of these studies have shown correlations between the amount of peritumoral edema and survival [3,6].

The topographic location of the tumors has been described as a potential factor affecting the outcome, particularly for tumor involvement in central brain structures, but the majority of the studies still use a lobar classification system [7,8,9]. One shortcoming of the standard lobar classification system is that if the tumor is invading subcortical regions between two lobes, this information risks not being included in the predictive models. The Brain-Grid system was created as a radiological overlay based upon anatomical landmarks that splits the brain into 48 voxels that may easily be associated with subcortical landmarks and white matter bundles. It has been used previously as a standardized subcortical classification system to emerging as an alternative the standard lobar classification system which neglect subcortical structures and landmarks [10]. This 3D coordinate system has been used successfully to distinguish predictive features in low grade gliomas but its application to glioblastoma has been limited [11]. Despite increasing interest in imaging biomarkers in glioblastoma, previous studies have primarily focused on tumor size, lobar location, or manually defined anatomical regions. The prognostic significance of quantitative subcortical tumor infiltration within a standardized anatomical framework remains largely unexplored. Furthermore, no previous study has, to our knowledge, combined fully automated MRI segmentation with the Brain-Grid classification system to evaluate volumetric biomarkers and voxel-specific tumor location in a molecularly homogeneous cohort of IDH-wildtype glioblastoma.

Therefore, the aim of this study was to determine whether quantitative subcortical tumor infiltration assessed using the Brain-Grid framework provides prognostic information beyond established preoperative factors such as age and tumor volume, while simultaneously evaluating the prognostic value of edema volume and edema-to-tumor ratio.

## 2. Materials and Methods

### 2.1. Patient Populations

All patient data included in the study was accessed through the U-CAN database. U-CAN is a Swedish nation-wide multi-center register and biobank containing clinical data and tissue samples for cancer research through collaborations between hospitals and universities [12]. In total it covers about one third of the Swedish population. We retrieved data of 341 patients (>18 years old) with histologically confirmed IDH-wildtype glioblastoma diagnosed between 2014 and 2023. We did not include patients that had undergone surgery at other hospitals than Uppsala University Hospital or patients where diagnostic MRI sequences were unavailable. We also chose not to include patients where a surgical biopsy was the sole surgical intervention, since the absolute tumor volume was not modified by the operation. We also included age at diagnosis, date of diagnosis, date of surgery, and date of death when applicable. Data access and subsequent analysis was carried out under approval of the local ethics committee (DNR: 2023-00876-01).

All patients were treated according to the Swedish national guidelines for brain tumors issued by the Regional Cancer Centers in collaboration (RCC).

### 2.2. Neuroimaging

**Figure 1:**
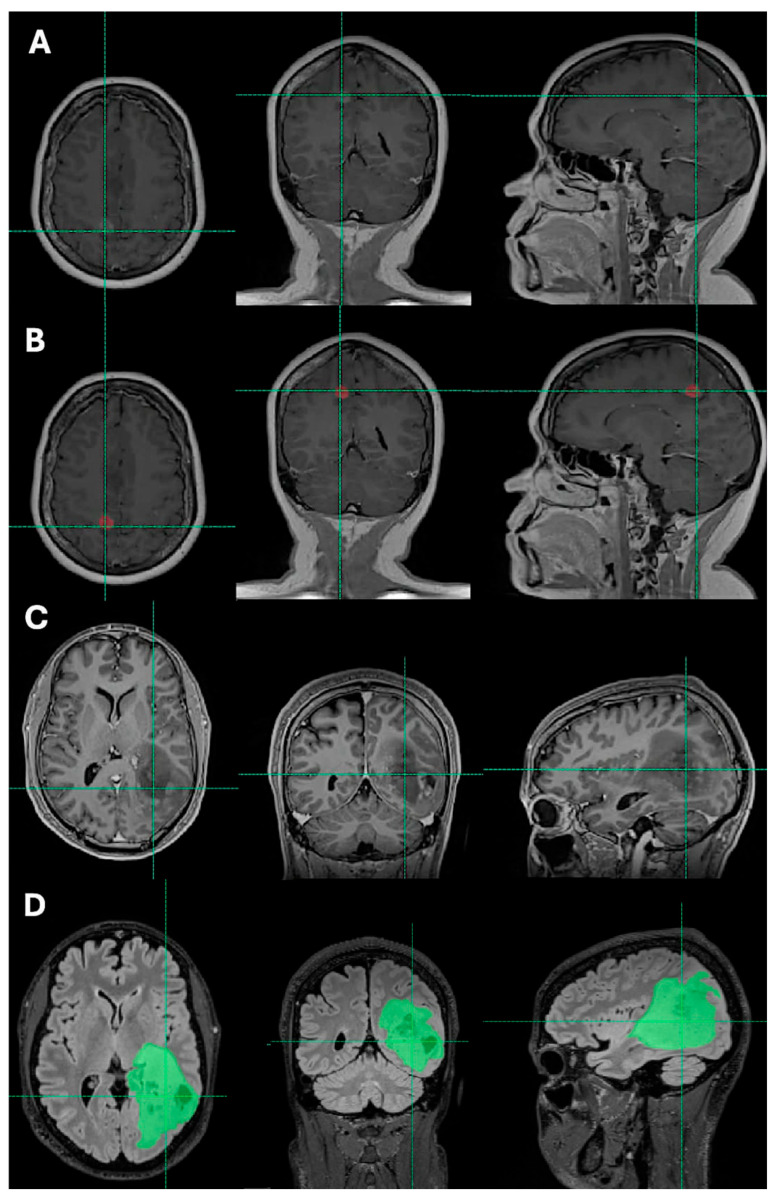
Sagittal, coronal and axial MRI scans from two patients in the study shown for conceptual purposes. Row (**A**) shows a patient with a right sided parietal tumor before automatic segmentation. Row (**B**) shows the same tumor after the automated segmentation procedure. Row (**C**) shows contrast-enhanced T1 images from another patient with a left-sided posterior temporal/occipital tumor. Row (**D**) shows FLAIR sequences from the same patient with the FLAIR signal highlighted in green, illustrating the peritumoral edema.

For each patient, the corresponding contrast-enhanced T1- and T2-FLAIR-MRI images of the time of diagnosis, as exemplified in Figure 1, were downloaded from the local hospital radiology server. We utilized Raidionics software (Version 1.3.0) to perform automatic segmentation of the tumor and the peritumoral edema on MRI [13]. We defined tumor volume, peritumoral edema and Brain-Grid voxels as variables of interest [14]. The segmentation results were visually inspected and all cases where the segmentation was inaccurate, or where the available images were not compatible with the software, were subsequently excluded. Since Raidionics software version could not compute the volume of edema it was calculated as T2 volume subtracted with contrast-enhancing volume on T1. Edema–tumor ratio (ETR) was then calculated as edema volume divided by tumor volume.

### 2.3. Statistical Analyses

#### 2.3.1. Volumetric Analyses

**Figure 2:**
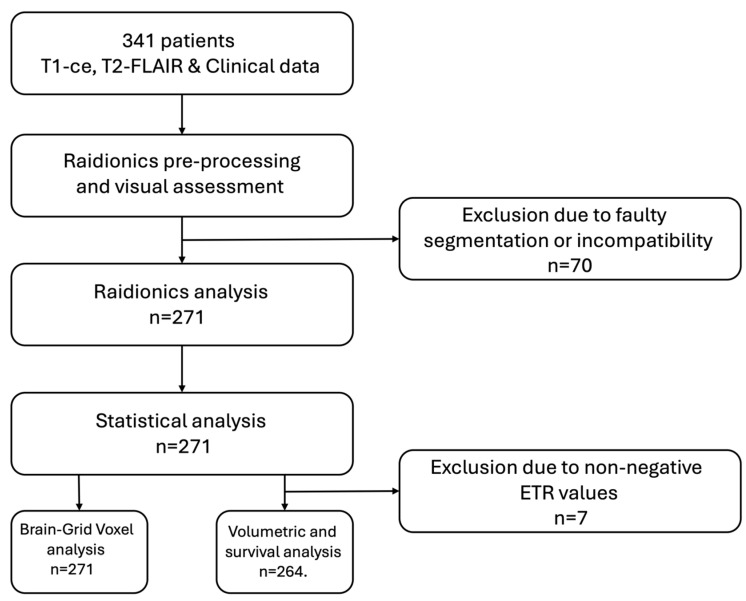
Conceptual overview of the imaging analysis processing. All cases where the automated segmentation failed or where the software was unable to process the images were excluded.

An overview of the entire analysis workflow is illustrated in Figure 2. Overall survival was calculated using Cox proportional hazards regression analysis. Survival time was defined as the interval from surgery to death or last follow-up with patients being alive at last follow-up treated as censored cases. Age, contrast-enhancing tumor volume, peritumoral edema volume and edema-to-tumor volume ratio were treated as continuous variables. Tumor volume, edema volume and ETR were log-transformed because of right-skewed distributions. Univariable Cox regression analyses were performed for each variable, followed by subsequent multivariate models adjusted for age and tumor volume. Hazard ratios with 95% confidence intervals were reported. We performed Kaplan–Meier and subsequent Log-Rank analysis of survival after performing a median split of edema volume and ETR respectively, dividing the patient cohort into two groups: high/low edema and high/low ETR. Overall survival was defined as the interval from the date of surgery to death from any cause or last documented follow-up. Univariable Cox proportional hazards regression analyses were first performed separately for each predictor variable. Variables of interest were subsequently evaluated in multivariable Cox proportional hazard regression models adjusted for age and preoperative tumor volume. Hazard ratios with 95% confidence intervals are reported.

#### 2.3.2. Quantification and Survival Analysis of Voxel Infiltration

Tumor infiltration of the Brain-Grid voxels was quantified by calculating the proportion of each voxel occupied by tumor after automatic image segmentation and spatial registration to the Brain-Grid atlas. Unlike previous Brain-Grid studies, which classified each voxel as simply infiltrated or non-infiltrated, the present study first quantified the percentage of tumor volume within every voxel before dichotomization for survival analyses.

Because very small overlap volumes may arise from minor image registration inaccuracies, interpolation artifacts during atlas transformation, or negligible tumor extension unlikely to represent clinically meaningful anatomical infiltration, a threshold of 5% tumor occupancy was applied. Voxels with <5% tumor occupancy were therefore classified as non-infiltrated, whereas voxels with ≥5% occupancy were classified as infiltrated. This threshold was selected to improve the robustness of the voxel classification rather than to represent a biological cut-off.

**Figure 3:**
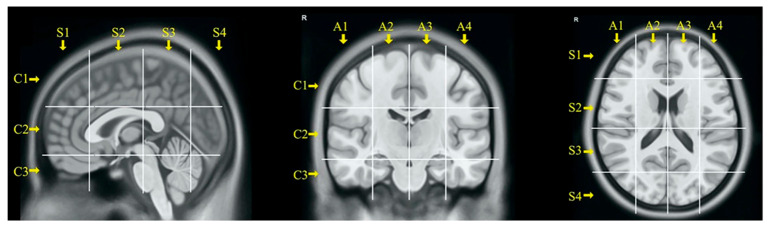
Principal layout of the Brain-Grid voxel system. The MRI scan is divided into 48 voxels representing the sagittal, coronal and axial plane. From Latini et al. [10]. R: right side.

To assess the overall extent of subcortical tumor infiltration, the total number of infiltrated Brain-Grid voxels (as shown in Figure 3), was calculated for each patient by summing all voxels classified as infiltrated. The resulting voxel count was treated as a continuous variable and analyzed using univariable Cox proportional hazards regression to evaluate whether increasing anatomical spread of tumor infiltration was associated with overall survival.

Voxel-specific survival analyses were subsequently performed to identify prognostically relevant anatomical regions. Only voxels infiltrated in at least 10 patients were included in these analyses to reduce unstable hazard estimates caused by sparse data. For each eligible voxel, patients were classified according to the presence or absence of tumor infiltration, and a separate multivariable Cox proportional hazards regression model was fitted with overall survival as the outcome. Each model included voxel infiltration as the predictor of interest and was adjusted a priori for age and preoperative tumor volume, which are established preoperative prognostic factors in glioblastoma. Hazard ratios (HRs) with 95% confidence intervals (CIs) were calculated for each voxel.

Because multiple voxel-wise comparisons were performed, *p*-values were adjusted using the Benjamini–Hochberg false discovery rate procedure to reduce the risk of false-positive findings. Statistical significance was defined as an adjusted *p*-value < 0.05.

All statistical analyses were performed in R studio (version 2025.09.2+418) running R version 4.4.0. We used scales 1.3.0, forcats 1.0.0, purr 1.0.2, string 1.5.1, tidyr 1.3.1, janitor 2.2.1, readr 2.1.5, broom 1.0.8, survminer 0.5.0, ggpubr 0.6.0, ggplot2 3.5.2, survival 3.8.3, dplyr 1.1.4 and readxl 1.4.5 as attached packages.

## 3. Results

**Figure 4:**
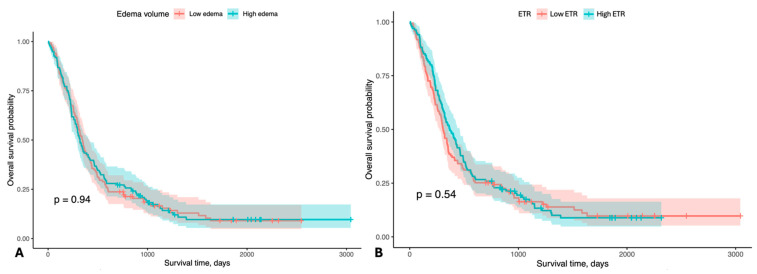
Kaplan–Meier curves illustrating the overall survival of the low and high edema groups (**A**) and the low and high ETR groups (**B**). No significant difference in overall survival could be seen between the groups.

Of the 271 subjects that were processed through the Raidionics pipeline, 235 cases (86.7%) were dead and 36 cases (13.3%) were censored. Mean age at time of diagnosis was 67 years (IQR 59–73), and mean survival time was 333 days (IQR 193–710). The median follow-up among censored patients was 1163 days. Mean contrast-enhancing tumor volume was 24.4 mL (IQR 28.9–46.5), mean edema volume was 57.4 mL (IQR 28.9–104.9) and mean ETR was 2.76 (IQR 1.41–5.59). Seven cases were excluded from the ETR analyses because of non-positive ETR values due to T1 contrast-enhancing volume equaling or exceeding the corresponding FLAIR volume. Univariable Cox proportional hazards analysis showed significant association between age and overall survival (*p* = 0.00016, HR 1.028, 95% CI 1.013–1.043) but no significant associations between log-transformed tumor volume, edema volume or ETR, and overall survival. Multivariate Cox proportional hazards analysis showed no significant associations between edema volume and overall survival when adjusted for tumor volume and age, nor did we find any significant associations between overall survival and log-transformed tumor volume or ETR when adjusted for age and ETR, and age and tumor volume respectively. Kaplan–Meier and Log-Rank analysis of high/low edema volume and high-low ETR groups showed no significant difference in overall survival, as illustrated in Figure 4.

### Voxel Infiltration Analysis

Infiltration in two Brain-Grid voxels (A3C2S2, *p* = 0.001, FDR-adjusted *p* = 0.022 and A3C2S1, *p* = 0.003, FDR-adjusted *p* = 0.047) was significantly associated with shorter overall survival. These voxels were infiltrated in 55% and 25% of cases respectively. No voxels were found to be associated with a better prognosis. Univariate Cox regression analysis showed no association between number of infiltrated voxels and overall survival, neither did multivariate Cox regression analysis adjusted for age and tumor volume. All voxels with at least 10 instances of infiltration are shown in Table 1.

## 4. Discussion

To our knowledge, this is the first study to combine automated MRI segmentation with voxel-wise Brain-Grid analysis in a large cohort of patients with IDH-wildtype glioblastoma. Using contemporary survival analyses with adjustment for censoring and multiple testing, we found that:

(1) The volume of the edema and the volume of the contrast enhancement were not correlated to OS in this cohort.
(2) Specific subcortical regions of the brain are associated with worse OS.
(3) Age is confirmed to be an independent variable affecting OS in patients with GBM.

### 4.1. Peritumoral Edema Analysis

Neither edema volume nor edema-to-tumor ratio demonstrated an independent association with overall survival in either univariable or multivariable Cox regression analysis. These findings suggest that the volumetric FLAIR-derived compartment used in this study is not an independent prognostic marker. However, because FLAIR hyperintensity represents a mixture of vasogenic edema and infiltrating non-enhancing tumor, these results should not be interpreted as evidence that biologically defined edema lacks prognostic importance.

These findings are contrary to those of a recent study exploring radiological features and survival, where the authors studied two datasets of glioblastoma patients using another fully automated segmentation and found edema to be the sole significant radiological feature for predicting survival [6]. On the other hand, studies such as recently published by Fang et al. explored the effect of the so called edema index (IE), which is another way of measuring edema and its proportion to tumor volume [15]. Amongst 164 patients they found no significant correlation between IE and OS, but between IE and progression free survival (PFS). We suggest that the peritumoral edema should be considered multifactorial, and that by simply analyzing it as one variable, other potentially relevant information may be overlooked. We thus conclude that a deeper understanding of the of cause and biological consequence of the edema itself is necessary to distinguish and correctly stratify its potential prognostic value.

### 4.2. Tumor Volume

The relevance of tumor volume as a prognostic factor in patients with glioblastoma is not fully elucidated and subject to debate. We could not find significant correlations between preoperative tumor volume and overall survival in the present study. Tumor volume has been recently suggested as a prognostic marker in conjunction with other factors, but not by itself [16]. A recent study by Johnstad et al. further analyzed tumor size and shape without finding significant correlations to overall survival, which is somewhat in concordance with our findings [17]. While it could be considered fairly intuitive to consider tumor volume as indicative of tumor severity, glioblastomas are a particularly difficult entity since a significant part of the biologically active tumor is diffusively infiltrating its surroundings and consequently does not show up on standard MRI scans [18]. Our results may support the idea that the importance of tumor volume is not mainly related to the preoperative volume, but rather to the residual tumor volume as proposed by the RANO resect group [19].

### 4.3. Brain-Grid Voxel Analysis

**Figure 5:**
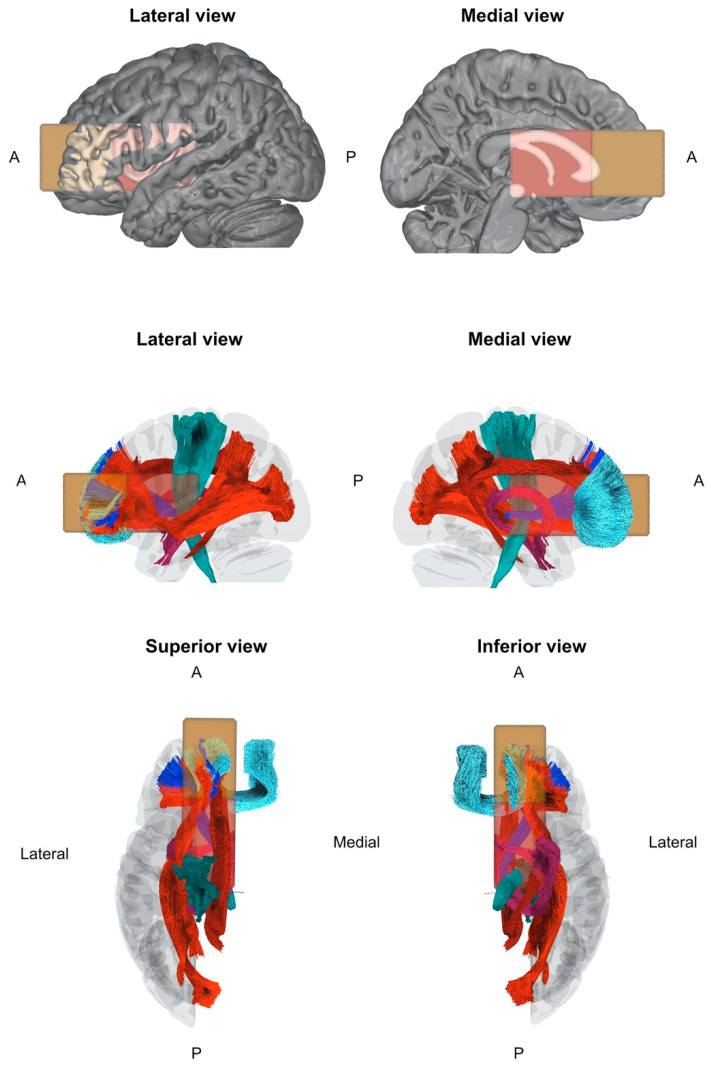
The image illustrates the position of the two Brain-Grid voxels (A3C2S1—tangerine, A3C2S2—red) associated with shorter OS. In the first row they are displayed on a 3D reconstruction of the left hemisphere to show the position within frontal lobe and basal ganglia. In the second and third rows they are displayed with the reconstructed white matter bundles according to the Brain-Grid tractography atlas on the sagittal plane in both lateral and medial view and on the axial plane in both superior and inferior view. The reconstructed bundles discussed in the text are: red—the inferior fronto-occipital fasciculus; dark blue—the anterior thalamic radiation; dark red—the cingulum; teal—the corticospinal tract; maroon—the fornix; light blue—the genu of corpus callosum. A: anterior; P: posterior.

The principal finding of this study is that infiltration of two centrally located Brain-Grid voxels (A3C2S1 and A3C2S2) remained independently associated with shorter overall survival after adjustment for age and preoperative tumor volume. Importantly, after correction for multiple testing, only two adjacent voxels remained significantly associated with overall survival, highlighting the importance of accounting for false-positive findings in voxel-wise analyses. Rather than representing isolated anatomical regions, these neighboring voxels represent a continuous left frontal–subcortical network, as illustrated in Figure 5. Voxel A3C2S1 encompasses the subcortical frontal white matter adjacent to the anterior cingulate cortex and genu of the corpus callosum, together with portions of the anterior thalamic radiation and inferior fronto-occipital fasciculus. Immediately posterior, voxel A3C2S2 encompasses the basal ganglia and internal capsule containing the anterior thalamic radiations and corticospinal tract beneath the insular cortex. Collectively, these interconnected structures contribute to executive function, attention, initiation of behavior, language, motor planning and motor execution [10,20,21,22,23]. Their close anatomical continuity suggests that the observed prognostic association reflects disruption of an integrated left frontal–subcortical network rather than isolated involvement of a single tract or nucleus.

The association between infiltration of this network and reduced overall survival is likely multifactorial. From a biological perspective, glioblastoma infiltration of these interconnected pathways may disrupt networks responsible for executive function, attention, language, motivation, motor planning and motor execution, leading to progressive neurological dysfunction and reduced functional reserve independently of tumor size. From a surgical perspective, the proximity of these regions to eloquent white matter tracts and deep gray matter nuclei may limit the extent of maximal safe resection because of the increased risk of permanent neurological deficits, thereby increasing the likelihood of residual tumor [24]. Thus, the observed survival disadvantage probably reflects both the intrinsic biological consequences of tumor infiltration within highly connected functional networks, and the therapeutic limitations imposed by their anatomical location. Notably, the fact that only these two anatomically adjacent voxels remained significantly associated with overall survival after correction for multiple testing further supports the interpretation that the prognostic effect reflects involvement of a continuous functional network rather than isolated anatomical structures. Although this interpretation remains hypothetical, it is consistent with current understanding of frontal–subcortical circuitry and supports the concept that precise subcortical tumor location provides prognostic information beyond conventional volumetric imaging measures.

In this study, no significant association was found between the total number of infiltrated Brain-Grid voxels and overall survival. This contrasts with the findings reported for low-grade gliomas by Latini et al. [11]. A possible explanation is the fundamentally different biological behavior of glioblastomas compared with other diffuse glial tumors. Lower-grade gliomas typically exhibit slow infiltrative growth, allowing time for cortical reorganization and functional compensation, and the anatomical extent of tumor infiltration therefore more closely reflects disease burden [11]. In contrast, glioblastomas are characterized by rapid proliferation and diffuse invasion along white matter pathways, frequently extending beyond the radiologically visible lesion through microscopic infiltration while demonstrating limited opportunity for neuroplastic adaptation [25,26,27]. Consequently, the number of radiologically infiltrated Brain-Grid voxels may not accurately represent the true extent of tumor invasion or biological aggressiveness. Furthermore, MRI-based segmentation of the contrast-enhancing tumor is unlikely to capture the entire infiltrative component of glioblastoma. Therefore, the number of positive Brain-Grid voxels probably underestimates the true extent of tumor infiltration to a greater extent than in lower-grade gliomas, where FLAIR abnormalities more closely correspond to infiltrative tumor growth [11].

### 4.4. Patient Age

Age was one of the predictive factors observed in this study with a significant correlation to OS. This is in line with the established consensus reflecting the increased prevalence of comorbidities and decreased tolerance to neurological symptoms and oncological treatment associated with higher age [7]. This study further concludes this correlation, confirming that age should be a factor alongside others such as performance status when planning patient treatment.

### 4.5. Strengths and Limitations

The strengths of this retrospective cohort study include the large sample size (n = 271) and the demonstration of the potential feasibility of automated analysis of radiological features. These factors contribute to a robust set of data with low risk of human error and/or bias in the extraction of radiological data and indicates the potential role of automated imaging processing pipelines in future clinical tumor prognosis assessment. A potential weakness of the study is the analysis of a relatively high number of variables which increases the risk of alpha errors.

As Raidionics software version that we used was unable to calculate edema volume, we synthesized this volume by subtracting the contrast-enhancing volume on T1 sequences from the high-signaling volume on T2 sequences. As the T2 sequences may theoretically contain non-enhancing tumor, there is a potential risk of underestimating tumor volume, which in turn could influence the ETR. There is also a risk of selection bias due to the exclusion of cases due to failed automated segmentation, image incompatibility, or biopsy-only management. Deep-seated, hemorrhagic, highly necrotic, or technically challenging tumors may be overrepresented among excluded cases. Similarly, biopsy-only cases are more likely to occur in eloquent or surgically inaccessible locations, which could potentially affect the observed voxel-specific survival patterns. We also acknowledge that further research is warranted to validate this indirect method of calculating edema volume against manual segmentation.

This study is based on a patient cohort spanning 10 years (2014–2023), during which the glioblastoma WHO classification system changed from WHO 2016 to WHO 2021. All patients included in the study were identified through the U-CAN biobank and were classified as IDH-wildtype glioblastoma according to the neuropathological diagnosis available at the time of inclusion. Histopathological and molecular assessments were performed within the framework of routine clinical practice at our institution. Consequently, molecular markers incorporated into the WHO 2021 definition of glioblastoma (e.g., TERT promoter mutation, EGFR amplification, or combined chromosome 7 gain/chromosome 10 loss) were not necessarily assessed uniformly across the entire study period. Thus, we suggest that future studies based on the uniformly applied WHO 2021 molecular criteria could provide greater diagnostic consistency.

This study lacks data about other potentially confounding patient factors such as performance status, MGMT methylation and extent of resection The extent of resection (EOR) is one of the strongest established prognostic factors in glioblastoma and represents a potential mediator and/or confounder of the observed association between tumor location and overall survival. Unfortunately, reliable and standardized EOR data were not available for the entire cohort and could therefore not be incorporated into the present analyses. Importantly, our study was designed to investigate preoperative prognostic imaging features available at the time of diagnosis. Since EOR is determined after surgery and is itself strongly influenced by tumor location, it may represent part of the causal pathway linking anatomical location to outcome. Consequently, adjustment for EOR could potentially attenuate location-related effects by controlling for a mediator rather than a purely independent confounder.

The role of MGMT methylation status in prognosticating glioblastoma survival is not fully understood. Several factors contribute to the somewhat uncertain prognostic value of methylation status, such as ambiguous retrospective results and the fact that it does not reliably correlate with other molecular markers such as IDH, ATRX and p53 [28]. There is also not a globally standardized method of assessing MGMT methylation status, and MGMT methylation status does not always translate predictably to protein expression levels and thus does not always predict individual response to temozolomide [29]. Several studies have investigated possible correlations between MGMT methylation status and tumor size and/or location. MGMT methylation status has been tentatively linked to cortical tumor location, but not to any specific anatomical region or voxel in the brain. [8] Preoperative tumor size has also been shown not to correlate with MGMT methylation status [30]. Thus, we argue that the lack of MGMT methylation data in our cohort does not necessarily pose a weakness, as MGMT methylation status is not a sole independent predictor. We acknowledge that future studies should aim to integrate MGMT methylation data in radiologic analyses to uncover any hitherto unknown biologically significant correlations.

## 5. Conclusions

This study demonstrates the feasibility of standardized quantitative assessment of subcortical tumor infiltration using the Brain-Grid framework to identify anatomically specific regions associated with overall survival beyond conventional volumetric imaging measures. These findings support the use of reproducible atlas-based imaging analyses for future prognostic modeling and provide a foundation for external validation in prospective multicenter cohorts.

## Figures and Tables

**Table 1:** Infiltration pattern characteristics and hazard ratios (HR), 95% CI, *p*-values and FDR-adjusted *p*-values for all voxels with at least 10 infiltrated cases. The two voxels that showed correlation with overall survival are displayed in bold. n: number.

Voxel	Infiltrated (n)	Non-Infiltrated (n)	HR	95% CI	*p*-Value	FDR-Adjusted *p*
**A3C2S2**	55	215	**1.71**	**1.25–2.34**	**0.001**	**0.022**
**A3C2S1**	25	245	**1.88**	**1.23–2.86**	**0.003**	**0.047**
A2C2S2	69	201	1.42	1.04–1.93	0.025	0.168
A3C2S4	16	254	0.52	0.28–0.95	0.035	0.168
A2C2S1	28	242	1.57	1.03–2.40	0.035	0.168
A3C1S3	22	248	0.59	0.36–0.97	0.036	0.168
A4C2S1	10	260	1.93	1.01–3.69	0.047	0.186
A4C3S2	43	227	0.71	0.50–1.03	0.071	0.249
A4C2S2	58	212	1.29	0.94–1.76	0.115	0.358
A1C2S3	50	220	0.76	0.54–1.08	0.132	0.369
A4C1S3	15	255	0.72	0.40–1.30	0.280	0.714
A4C2S4	12	258	0.73	0.39–1.39	0.340	0.788
A1C2S1	13	257	1.30	0.73–2.31	0.366	0.788
A3C1S2	19	251	1.23	0.75–2.04	0.409	0.818
A3C2S3	38	232	0.87	0.59–1.27	0.474	0.828
A4C1S2	12	258	1.23	0.67–2.26	0.510	0.828
A1C1S3	19	251	0.85	0.50–1.43	0.536	0.828
A1C1S2	14	256	0.83	0.46–1.50	0.543	0.828
A1C3S2	33	237	1.12	0.75–1.66	0.587	0.828
A2C3S2	14	256	0.85	0.46–1.57	0.611	0.828

## Data Availability

The data presented in this study are available on request from the corresponding author.
